# Invasive goldfish trigger a regime shift in experimental lake ecosystems of varying trophic state

**DOI:** 10.1111/1365-2656.70259

**Published:** 2026-04-27

**Authors:** William D. Hintz, Hannah Barrett, Rick A. Relyea

**Affiliations:** ^1^ Department of Environmental Sciences and Lake Erie Center The University of Toledo Oregon Ohio USA; ^2^ Department of Biological Sciences, Darrin Fresh Water Institute Rensselaer Polytechnic Institute Troy New York USA; ^3^ Johnny Morris Institute of Fisheries, Wetlands, & Aquatic Systems University of Missouri Columbia Missouri USA

**Keywords:** alien species, biodiversity, carp, conservation, ecosystem collapse, exotic pets, food web

## Abstract

1. The ongoing pressure of biological invasions caused by the pet trade continues to restructure the earth's ecosystems. The release of pets and ornamentals has altered species interactions, ecosystem functions, spread disease and rewired food webs.
2. We assessed the effects of a high‐risk, yet understudied invasive species—that is, goldfish, *Carassius auratus*—on experimental freshwater ecosystems by integrating additive and substitutive experimental designs across two trophic states (i.e. oligotrophic vs. eutrophic). We hypothesized that goldfish would trigger a regime shift characteristic of more algae, reduced water quality and negative impacts on native species across multiple trophic levels.
3. Under eutrophic conditions, goldfish induced a rapid shift in mesocosm state characterized by increased suspended solids and reduced water clarity; however, increases in phytoplankton and reductions in filamentous algae were largely driven by total fish density (native or invasive), consistent with strong niche overlap between the two species. Through consumption and habitat loss, goldfish caused reductions in snails, amphipods and zooplankton. Goldfish also reduced native fish condition, likely through exploitative competition.
4. Our results indicate that goldfish are undesirable for both oligotrophic and eutrophic lakes, although some impacts will depend on trophic state. These results suggest that natural resource managers worldwide should consider management action to reduce or prevent goldfish invasions. Despite many viewing goldfish as signs of prosperity, luck and wealth, it is critically important to inform the public that their pets can grow into sizable pests that will harm freshwater ecosystems.

## Graphical abstract

The pet trade distributes exotic animals around the globe that enrich human lives. However, some pets become pests when released into the wild. This study provides experimental evidence that goldfish will negatively affect native freshwater organisms and ecosystems. The results support the need for management action to reduce or prevent goldfish invasions that cause a dramatic restructuring of freshwater ecosystems.

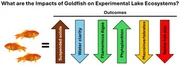

## 1 INTRODUCTION

Biological invasions reduce biodiversity, which in many cases might not be reversible (Jeschke et al., 2014; Ricciardi, 2007; Ricciardi et al., 2013; Ricciardi & Rasmussen, 1999). One mechanism by which non‐native species have spread to ecosystems around the world is via the multibillion dollar pet trade (Dickey et al., 2023; Gippet & Bertelsmeier, 2021). The release of pets and ornamentals has triggered losses of biodiversity in terrestrial and aquatic ecosystems worldwide by altering species interactions, ecosystem functions, spreading disease and rewiring food webs (Gallardo et al., 2016; Lockwood et al., 2019; Mollot et al., 2017; Tilmans et al., 2014). Unfortunately, policies related to the pet trade might only be making the problem worse by increasing unwanted introductions rather than preventing them (Patoka et al., 2018). This persistent pressure of introduced species has and will continue to cause a major restructuring of the earth's ecosystems.

One of the most widely recognized ornamental species of the pet trade is the goldfish (*Carassius auratus*; e.g. Strecker et al., 2011). They have been domesticated for over 1000 years, transported around the world for centuries and are one of the most important farmed fish globally (Chen et al., 2020). They were introduced to European countries as early as the 1600s and North America in the mid‐1800s (Copp et al., 2010; Nico et al., 2025). In addition to being pets, goldfish are widely used in commercial or residential ponds in an attempt to control several types of algae. While considered symbols of prosperity, luck and wealth in some cultures, the intentional release of goldfish has led to undesirable, undetected and often ignored invasions around the globe (Copp et al., 2010; Massey et al., 2025). Media attention towards goldfish has increased over the last couple decades and citizen‐science accounts of goldfish presence have increased exponentially over the last decade (Massey et al., 2025). However, policies often overlook the goldfish as an invasive species (Copp et al., 2010). A recent review highlights the surprising lack of information we have on the impacts of goldfish among freshwater ecosystems (Massey et al., 2025), which prevents establishing sound policies and management. Thus, it is important to explore the ecological consequences of goldfish invasions among a broad spectrum of environmental conditions.

Current evidence suggests that goldfish are a high‐risk, high‐impact invader in freshwater ecosystems globally. This is due to their generalist diet, feeding rate and mode of feeding where they churn up the benthos and resuspend sediments (Dickey et al., 2022, 2023; Monello & Wright, 2001; Richardson et al., 1995). Goldfish are related to other invasive carps (Chen et al., 2020; Massey et al., 2025) and can reach lengths of 45 cm and live 30 years or more in natural ecosystems (Brown et al., 2018). They can thrive in a wide range of environmental conditions, from low‐nutrient, clear water to high‐nutrient, low‐oxygen, highly turbid waters (García‐Berthou et al., 2005; Massey et al., 2025; Savini et al., 2010). In fishless permanent ponds, goldfish restructure food webs (Lejeune et al., 2024). Such ponds move from a macrophyte‐ and clear water‐dominated state to a phytoplankton‐dominated community with an overabundance of detritus and nutrients; the food web became simplified with lower taxonomic and functional diversity, which was linked to a shift in primary producer dominance (Lejeune et al., 2024). Goldfish also trigger trophic cascades by reducing zooplankton abundance and increasing phytoplankton abundance while negatively affecting benthic macroinvertebrates and macrophytes directly (Massey et al., 2025). Much of the knowledge on the ecological impacts of goldfish come from fishless ponds or field studies where effects of the goldfish can be confounded with other stressors (Massey et al., 2025). However, goldfish also are found in a diversity of lakes containing fish, including some of the world's largest lakes (e.g. Boston et al., 2024). It is important to assess the impacts of goldfish in these ecosystems with diverse ecological communities and food webs, which could respond much differently than fishless pond communities.

In freshwater ecosystems, the impact of invasive species can vary with trophic state (i.e. nutrient availability). Nutrient availability can mediate the bottom‐up and top‐down effects of invasive species and their interactions with native competitors and predators (Hintz et al., 2019; Preston et al., 2018; Schuler et al., 2020). Many invasive species can proliferate more quickly in eutrophic (i.e. nutrient‐rich) waters because the larger pool of energy can enhance the rates of individual growth, reproduction and population growth (Strayer, 2010; Strayer et al., 2004). In oligotrophic (i.e. low‐nutrient) waters, the pool of energy is limited, which can suppress population growth and perhaps limit the ecological impacts of invasive species (Elser et al., 2007; Strayer, 2010). Regardless of trophic state, there are numerous examples of invasive species altering the outcomes of predator–prey dynamics, competing with native species, triggering changes in nutrient cycling and modifying habitat in oligotrophic systems. Thus, the interaction between trophic state and the impacts of invasive species highlights the importance of context‐specific ecological information needed for management strategies and policy.

We used an experimental ecosystem approach to understand the effects of invasive goldfish across different trophic states. Our experimental ecosystem contained a food web with representatives from major trophic levels common to lakes. We manipulated native fish and invasive goldfish by combining an additive and substitutive experimental design to enhance our ability to draw inference about the impacts of invasive species in oligotrophic and eutrophic conditions. Our goal was to test the hypothesis that goldfish would trigger a regime shift from a clear to a turbid state that was different than a native fish species that is found throughout North America. We broadly defined an ecological regime shift as an abrupt change leading to a rapid reconfiguration of our experimental ecosystem triggered by goldfish when compared to a native community (Andersen et al., 2009). We examined abiotic and biotic responses to test whether goldfish (1) reduce water clarity, (2) increase phytoplankton but reduce filamentous macroalgae and periphyton, (3) reduce the density of major zooplankton taxa and benthic macroinvertebrates and (4) reduce the condition of the native fish species. We further hypothesized that a eutrophic system would magnify the effect sizes of these responses—that is, greater reductions or increases relative to the native fish treatments.

## 2 METHODS

### 2.1 Experimental design

**FIGURE 1:**
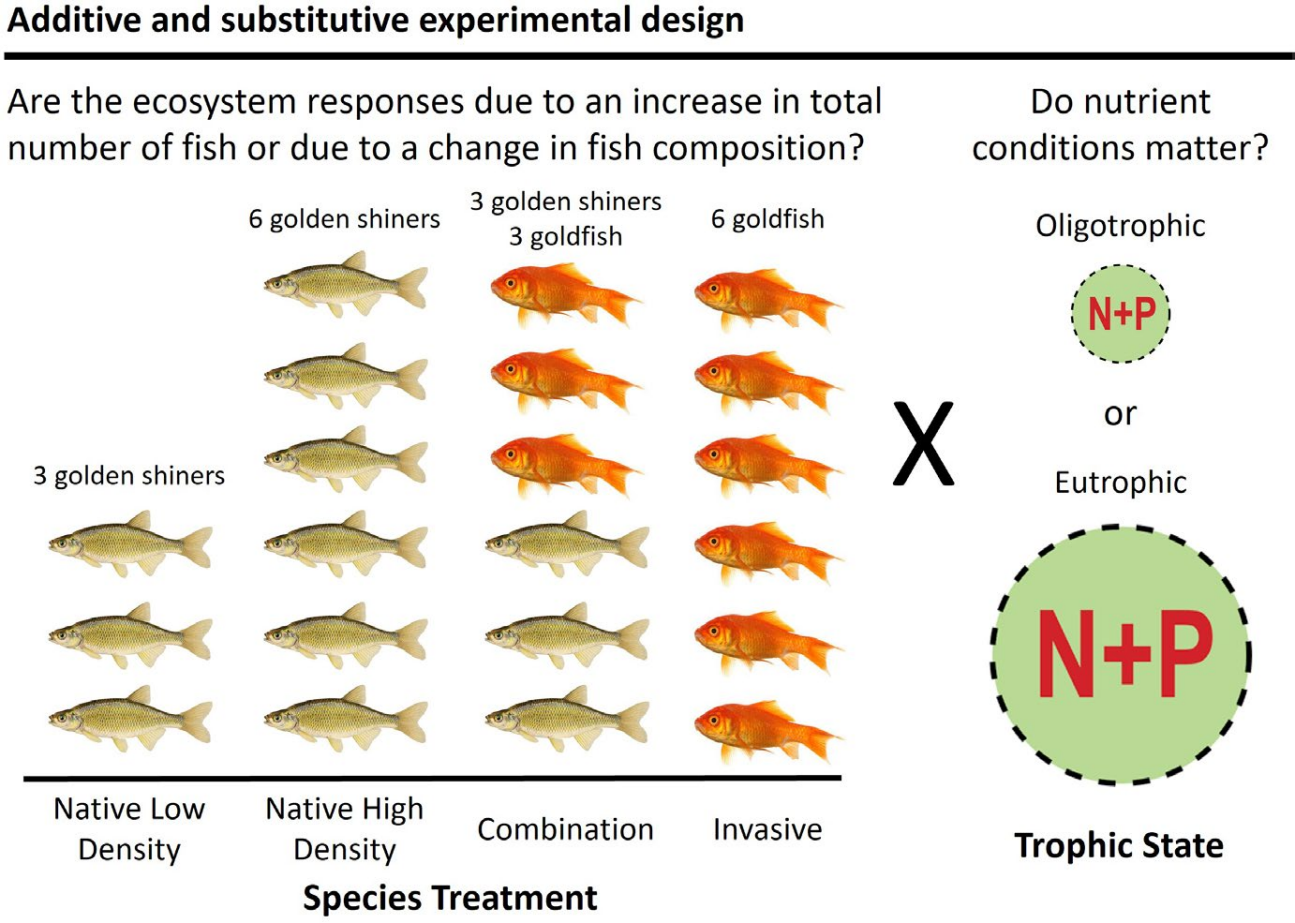
Additive and substitutive experimental designs combined to understand the impacts of the invasive goldfish on an experimental freshwater ecosystem. The native‐low and native‐high treatments contained three and six native shiners, respectively. The combination treatment contained three shiners plus three goldfish, and the invasive treatment contained six goldfish. These four species treatments were crossed with nutrient concentrations representing oligotrophic and eutrophic conditions. The eight treatment combinations were replicated four times.

When testing the effects of invasive species, additive designs often limit inference about whether a greater density of individuals affected ecosystem responses, but are useful for studying pre‐ and post‐invasion responses. In contrast, substitutive designs can limit inference about responses at different densities pre‐ and post‐invasion, but are useful for understanding how species composition affects invasion responses. We combined these designs to enhance our ability to answer whether the changes in our experimental ecosystem were due to a higher total density of fish or due to a change in species composition of fish by adding the invasive species. Thus, we crossed four fish treatments and two nutrient conditions to understand how goldfish affected freshwater food webs (Figure 1).

We used the golden shiner (*Notemigonus crysoleucas*) as our native fish species in the experiment because this species often overlaps with the distribution of introduced goldfish populations (Nico et al., 2025; Nico & Neilson, 2025). There is also intense habitat and diet overlap between the golden shiner and goldfish—both are opportunistic omnivores consuming freshwater plants, macroalgae, phytoplankton, zooplankton and macroinvertebrates (Hensley & Courtenay, 1980; Moyle, 2002; Murdy et al., 1997; Page & Burr, 2011). The four fish treatments were a low density and a high density of shiners (three or six individuals), a species mixture (three shiners + three goldfish) and a high density of goldfish (six goldfish). We crossed these four treatments with two nutrient treatments representative of oligotrophic lakes and eutrophic lakes because invasive species can have very different effects among freshwater ecosystems of varying trophic state (Byers, 2002; Gallardo et al., 2016; Hintz et al., 2019).

Our eight treatments were replicated four times and assigned randomly to 32 experimental mesocosms (volume = 1210 L). We filled each mesocosm with water hauled from Lake George in the Adirondack region of New York, United States. Lake George is a large and deep oligotrophic lake. A detailed, multi‐decade study on the water chemistry and quality of Lake George can be found in Hintz et al. (2020). This water served as our ‘ambient’ water treatment with no nutrient addition. For the high‐nutrient treatments, we augmented water from the oligotrophic lake with 1.15 mg N L^−1^ as ammonium nitrate (NH_4_NO_3_) and 0.05 mg P L^−1^ as sodium phosphate dibasic anhydrous (Na_2_HPO_4_). We added nutrients at a ratio of 23:1 total nitrogen (TN):total phosphorus (TP) as this ratio is near the threshold of a typical eutrophic lake, but higher than a hypereutrophic or dystrophic lake (Downing & McCauley, 1992). We added nutrients weekly to maintain eutrophic conditions throughout the experiment as dissolved nutrients can be taken up rapidly (Plath & Boersma, 2001). We did not measure TN and TP during our experiment because the absolute concentrations likely changed each day until the next nutrient influx (Plath & Boersma, 2001) and depended on the development and dynamics of the ecological community. We also added 0.08 m^3^ of sand and 100 g of oak (*Quercus* sp.) leaf litter to each mesocosm to serve as substrate and a basal nutrient source for the experimental community.

**FIGURE 2:**
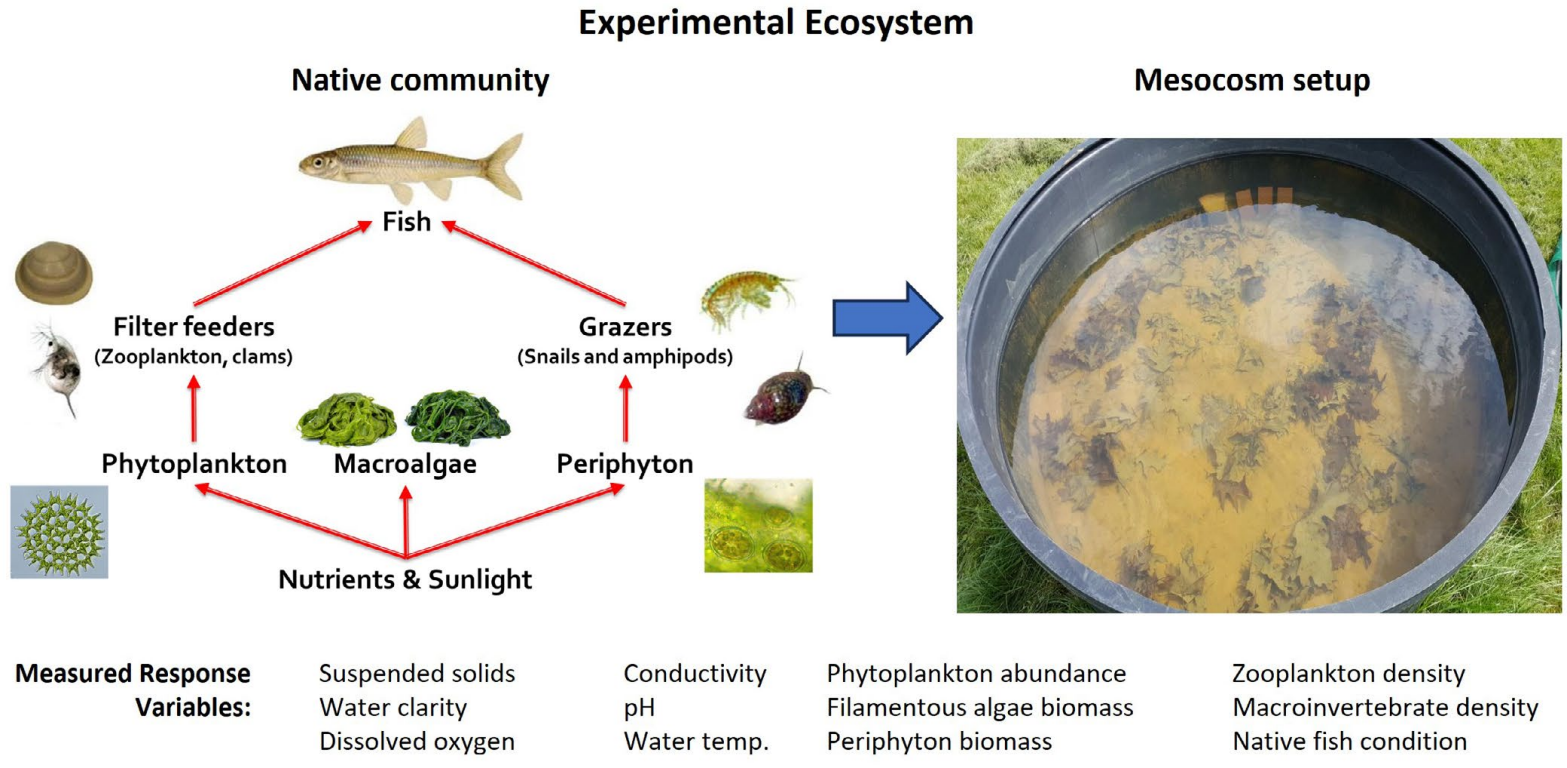
Our experimental freshwater ecosystem contained a native ecological community spanning multiple trophic levels where 12 variables were measured in response to four fish treatments and two nutrient treatments, which represented oligotrophic and eutrophic conditions.

We created an experimental community in each mesocosm consisting of organisms collected from local lakes. We chose representative species commonly found in lake ecosystems and among different trophic levels that included phytoplankton, periphyton, filamentous macroalgae, zooplankton, snails, amphipods, clams and fish (Figure 2). We collected vertical plankton tows (mesh = 64 μm) from Lake George and concentrated the contents into a 19‐L bucket. We mixed the 19 L of concentrated plankton well and added 0.5 L to each mesocosm to jumpstart the limnetic communities. We mixed the concentrated plankton community before adding to each mesocosm. We then added 10 *Physella acuta* snails and 20 *Hyalella* amphipods collected from Crystal Lake (42°38′33.80″ N, 73°33′7.05″ W), 20 *Gammarus* amphipods collected from Crooked Lake (42°36′45.12″ N, 73°31′29.64″ W) and 10 filter feeding *Sphaerium* pea clams from a private wetland (42°46′13.56″ N, 73°38′42.83″ W) in upstate New York (USA) to jumpstart the benthic macroinvertebrate community. The same number of snails, amphipods and clams were added to each mesocosm from each source to ensure the same starting community. We allowed the ecological community to develop for 35 days, after which we added the golden shiners and goldfish (purchased from Carolina Biological®) to their respective mesocosms. The experiment began on 27 July 2017 and ended on 28 September 2017 (duration = 61 days). We covered the mesocosms with 60% shade cloth, which allowed enough light to pass through for primary production but prevented terrestrial insects from colonizing the tanks through oviposition.

### 2.2 Quantifying experimental ecosystem responses

#### 2.2.1 Abiotic conditions

We used a calibrated YSI ProPlus Multiparameter Instrument (YSI, Inc., Yellow Springs, OH, USA) to measure water pH, conductivity (μS/cm), temperature (°C) and dissolved oxygen (mg O_2_ L^−1^). We measured non‐volatile suspended solids (NVSS) because many of the invasive ‘carps’ throughout the world are known to increase the suspension of inorganic matter due to their feeding methods (Pietsch & Hirsch, 2015). Similar to other studies, we quantified NVSS by filtering 1 L of water from each tank through pre‐weighed, 90‐mm Whatman® glass microfiber filters. We then ashed the filters in a furnace for 4 h at 550°C and re‐weighed the ashed filters to estimate the total inorganic matter (Kelly et al., 2018; Knowlton & Jones, 2000).

We measured light attenuation with a LI‐COR® Biosciences LI‐250A light meter by taking a ‘surface (*L*_s_)’ measurement (~1 cm under the water's surface) and a second ‘depth (*L*_D_)’ measurement (21 cm under the water's surface). Using these measurements, we calculated light attenuation (*K*_d_) for each mesocosm as follows:

$$K_{d}=\frac{\ln\left(\frac{L_{s}}{L_{D}}\right)}{0.2}$$

where 0.2 is the distance in m (20 cm) between the *L*_s_ and *L*_D_ measurements with the light meter (Arst et al., 1997). We interpreted higher light attenuation as a reduction in water clarity. Sampling for abiotic conditions occurred on 11 September.

#### 2.2.2 Primary producers

We quantified the abundance of phytoplankton, filamentous macroalgae and periphyton. We used the concentration of chlorophyll *a* to estimate phytoplankton abundance. We filtered 1 L of tank water through 1.2 μm Whatman® glass microfiber filters. The filters were kept frozen until we performed fluorometry with acid correction to determine chlorophyll *a* concentration of the samples (Arar & Collins, 1997). We collected periphyton and phytoplankton samples on 20 September.

For filamentous algae biomass, we mechanically removed all visible filamentous algae by hand from each tank on 22 September. We then squeezed excess water out of the bundle of filamentous algae, dried it for 10 days at 60°C and weighed the dried mass for each tank.

For periphyton biomass, we placed 7.6 × 15.2 cm ceramic tiles in each tank to serve as a standardized substrate. One tile was removed from each mesocosm, scrubbed and rinsed with RO water three times to generate a slurry. The slurry was filtered through pre‐weighed and pre‐dried (60°C for 24 h) glass microfiber filters, dried at 60°C for 48 h and then reweighed to determine periphytic biomass. We collected periphyton samples on 20 September.

#### 2.2.3 Zooplankton

We estimated the abundance of cladocerans, copepods and rotifers to understand responses of the limnetic grazer community. We submerged a 0.2‐L tube subsampler in all four cardinal directions and the centre of each mesocosm, filtered each subsample through 64‐μm Nitex screening and combined them together into a single 1‐L filtered sample for each mesocosm. We preserved each mesocosm sample in 2.5% acidified Lugol's solution for later enumeration under a microscope. We collected zooplankton samples on 20 September.

#### 2.2.4 Macroinvertebrates

We sampled macroinvertebrates on 22 September in two ways. First, we scraped 50% of the side wall area of each mesocosm with 250‐μm aquarium nets for snails. Second, we sampled 50% of the bottom area in all four cardinal directions by sweeping from the centre of the mesocosm to the side wall with a 500 μm D‐frame net to capture snails, clams and amphipods. All benthic sweeps and sidewall scrapes were combined into a single sample for each mesocosm and preserved in 70% ethanol for later enumeration.

#### 2.2.5 Native fish

We netted the goldfish and golden shiners with large‐mesh dipnets on 28 September. We euthanized all fish in MS‐222 under protocol number REL‐003‐15 (approved by Rensselaer Polytechnic Institute's Institutional Animal Care and Use Committee). The golden shiners were then rinsed, patted dry with paper towels, weighed (*W*, nearest g), measured (fork length FL, mm) and preserved in 70% ethanol. We assessed whether goldfish affected native fish size among the experimental treatments using Fulton's condition factor where fish condition = (*W*/*L* ^3^) × 10^5^ (e.g. *W* in grams, *L* in millimetres; Pope & Kruse, 2007). We averaged shiner mass and FL of each mesocosm. The mesocosm average served as replicates (i.e. individual fish were not replicates). We were unable to recover three of the 96 shiners used in the experiment, one in two high‐density mesocosms and one in a combination treatment; thus, average mass and FL were based on five shiners for the high‐density replicates and two shiners for the one combination replicate. No native fish were included in the high‐density goldfish treatment, so this treatment was excluded from our analyses of golden shiner size.

### 2.3 Statistical analyses

We quantified 11 ecosystem response variables—not including native fish size—in our experiment with the ecological responses having multiple species‐level or taxa‐related responses as described above (Figure 2). To identify community‐level shifts among our two experimental treatments (i.e. species and trophic state) and control the familywise error rate, we used multivariate analysis of variance (MANOVA). We use a MANOVA with all 11 response variables to assess if an ecosystem shift occurred among our experimental treatments. If we found main or interactive effects, we proceeded with a MANOVA for each of the major ecological groupings: abiotics (six responses), primary producers (three taxa‐level responses), zooplankton (three taxa‐level responses) and macroinvertebrates (four species‐level responses). Native fish condition was excluded from the MANOVA analyses because native fishes were excluded from the invasive‐species treatment (i.e. goldfish only). We performed MANOVA analyses in R (version 4.4.2; R Core Development Team, 2024).

If the MANOVAs revealed statistically supported differences in our response variables among our treatments and an interaction, we used two‐way analysis of variance (ANOVA) to test for statistically supported differences for the individual responses within the major ecological groupings. We use one‐way ANOVA if MANOVA revealed only a main effect of species treatment or trophic state on our response variables. We tested the assumptions of normality and equal variance using a Shapiro–Wilk test and Brown–Forsythe test, respectively. If the underlying assumptions were not met, we log_10_(*y*)‐transformed the data. If a response variable contained zeros, we used a log_10_(*y* + 1) transformation. If we found statistically supported interactions or main effects in the ANOVAs, we use a Holm‐Sidak post hoc test to control for the family‐wise error rate among the treatment comparisons. If we needed to use a log_10_ transformation, we graphed these results on a log_10_ scale to match the statistical analyses. Response variables requiring a log_10_ transformation were the taxa‐ and species‐level responses for the primary producers, macroinvertebrates and zooplankton. These transformations are shown on corresponding figure axes. If we did not transform the data, the data were graphed on the scale of the response variable. For statistically supported differences in our response variables among our treatment groups, we describe effect sizes as the percent (%) change between means on the scale of the response variable. We performed ANOVA analyses in SigmaPlot® 16.0.

### 2.4 Ethics approval

Approval to use fishes as part of this study was approved by Rensselaer Polytechnic Institute's Institutional Animal Care and Use Committee as part of protocol number REL‐003‐15.

## 3 RESULTS

We found a statistically supported interactive effect between the species treatment and trophic state on our ecosystem response variables (MANOVA: Pillai–Bartlet statistic = 2.4, *F*_48,33_ = 2.9, *p* = 0.001). Thus, we conducted separate MANOVAs on abiotic conditions, primary producers, zooplankton and macroinvertebrates to investigate the related ecosystem responses.

### 3.1 Abiotic conditions

We found an interactive effect between the species treatment and trophic state on our abiotic response variables (MANOVA: Pillai–Bartlet statistic = 1.1, *F*_18,63_ = 2.0, *p* = 0.027). We followed up with a two‐way ANOVA to determine the individual responses of NVSSs, water clarity, dissolved oxygen, conductivity, pH and temperature to species treatment and trophic state.

**FIGURE 3:**
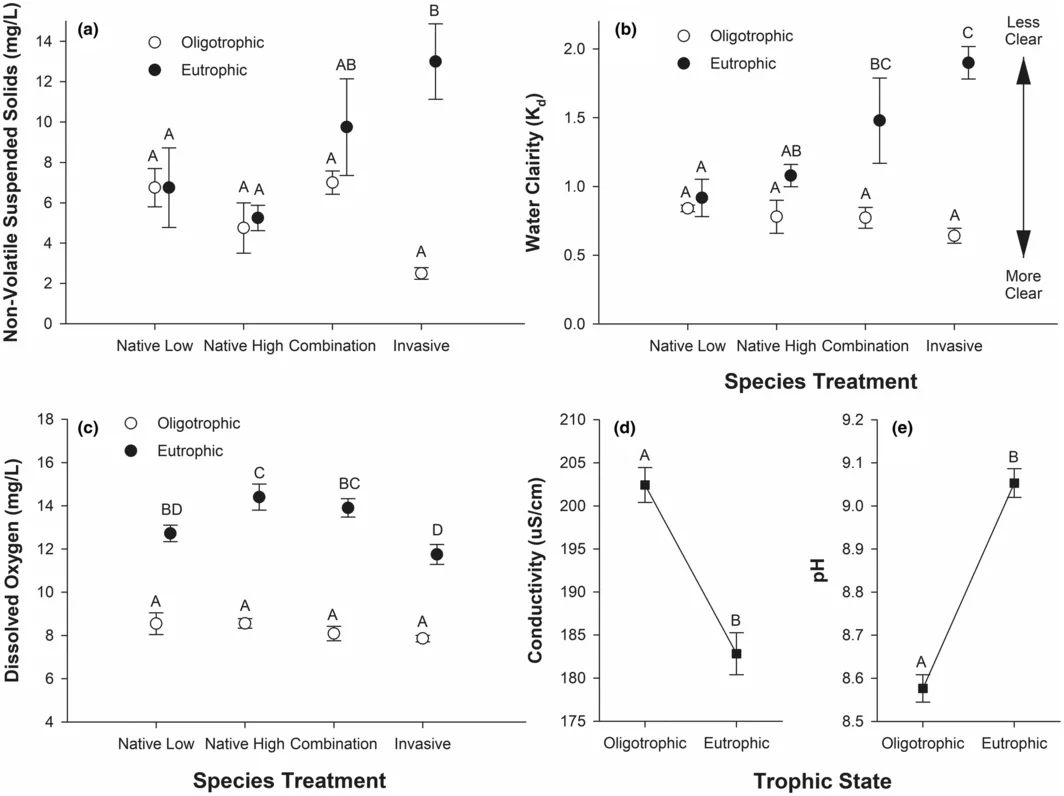
The interactive effects of species treatment and trophic state on abiotic conditions, including (a) non‐volatile suspended solids (NVSS), (b) water clarity (light attenuation, *K*_d_) and (c) dissolved oxygen, and the main effects of trophic state on (d) conductivity and (e) pH. All error bars are ±1 SE. Letters above error bars indicate statistical differences where *p* < 0.05 for Holm‐Sidak pairwise comparisons.

We found an interactive effect between species treatment and trophic state on NVSS (*F*_3,24_ = 5.6, *p* = 0.004). This interaction occurred because species treatment had no effect in oligotrophic conditions, but strong effects in eutrophic conditions. Goldfish increased NVSS by as much as 81% in eutrophic conditions (Figure 3a).

We found a similar interactive effect between species treatment and trophic state on light attenuation (*F*_3,24_ = 7.2, *p* = 0.001). Once again, species treatment had no effect in oligotrophic conditions, but strong effects in eutrophic conditions. Goldfish increased light attenuation by as much as 65% in eutrophic conditions (Figure 3b).

We found an interactive effect between species treatment and trophic state on DO (*F*_3,24_ = 3.2, *p* = 0.040). Species treatment had no effect under oligotrophic conditions. Under eutrophic conditions, however, the invasive treatment had 16% and 19% lower DO than the combination and native high‐density treatments, respectively. The DO in the low‐density native species treatment was 12% lower than the high‐density native species treatment in eutrophic conditions. Among all the species treatments, DO was 33%–42% lower in oligotrophic conditions compared to eutrophic conditions (Figure 3c).

We found no interaction or main effect of species on water conductivity (all *F* ≤ 2.3, all *p* ≥ 0.102), but we did find a main effect of trophic state on conductivity (*F*_1,24_ = 46.2, *p* < 0.001). Conductivity was 9% lower in eutrophic conditions compared to oligotrophic conditions (Figure 3d).

We found no interaction or main effect of species on pH (all *F* ≤ 2.3, all *p* ≥ 0.168). However, we found a main effect of trophic state on pH (*F*_1,24_ = 117.2, *p* < 0.001), with 4% lower pH under oligotrophic conditions compared to eutrophic conditions.

We did not find any interaction or main effects of our treatments on water temperature (all *F* ≤ 2.0, all *p* ≥ 0.162).

### 3.2 Primary producers

We found an interactive effect between the species treatment and trophic state on our abiotic response variables (MANOVA: Pillai–Bartlet statistic = 0.7, *F*_9,72_ = 2.2, *p* = 0.032). We followed up with a two‐way ANOVA to determine the individual responses of phytoplankton (chlorophyll *a*), filamentous algae and periphyton to species treatment and trophic state.

**FIGURE 4:**
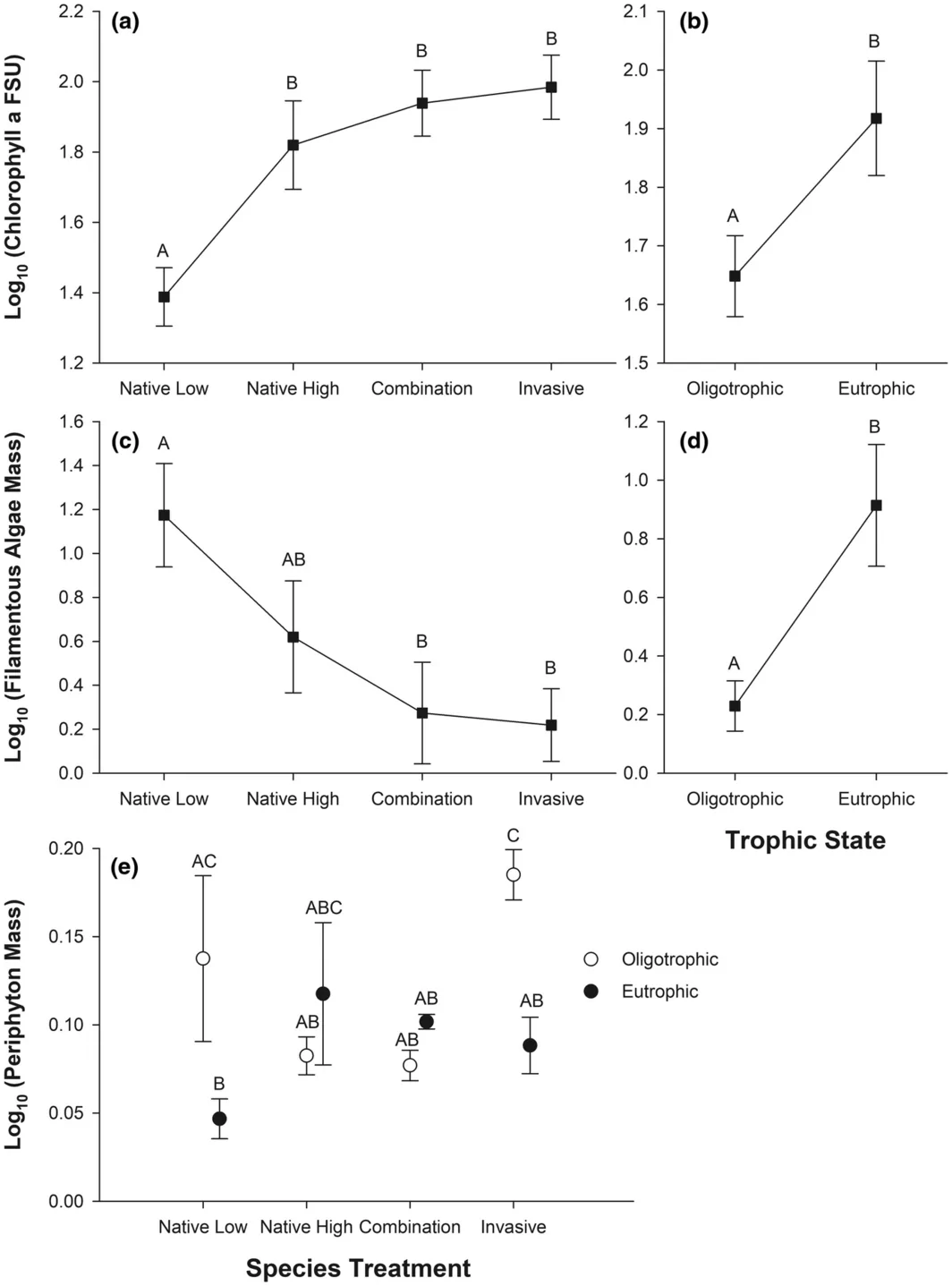
Main effects of species and trophic state on phytoplankton (a, b, measured as chlorophyll *a* concentration), filamentous algal biomass (c, d) and the interactive effects on periphyton biomass (e). All error bars are ±1 SE. Letters above error bars indicate statistical differences where *p* < 0.05 for Holm‐Sidak pairwise comparisons.

We found no treatment interaction on phytoplankton abundance (*F*_3,24_ = 1.2, *p* = 0.336), but there were main effects of species treatment (*F*_3,24_ = 9.9, *p* < 0.001) and trophic state (*F*_1,24_ = 9.7, *p* = 0.005). Phytoplankton abundance was 68%–76% lower in the low‐density native treatment compared to the high‐density native species, combination and invasive treatments (Figure 4a). Phytoplankton was 51% lower in oligotrophic conditions compared to eutrophic conditions (Figure 4b).

We found no treatment interaction on filamentous algae mass (*F*_3,24_ = 0.8, *p* = 0.525), but there were main effects of species treatment (*F*_3,24_ = 5.4, *p* = 0.006) and trophic state (*F*_1,24_ = 13.1, *p* = 0.001). Filamentous algae was 68% and 90% less abundant in the combination and invasive treatments, respectively, compared to the low‐density native treatment (Figure 4c). The mass of filamentous algae was 94% lower in oligotrophic conditions compared to eutrophic conditions (Figure 4d).

We found a treatment interaction on periphyton biomass (*F*_3,24_ = 4.4, *p* = 0.013). Under oligotrophic conditions, periphyton biomass was 60% and 64% lower in the native‐high density and combination treatments, respectively, compared to the invasive treatment (Figure 4e). The other three species treatments were not different from the low‐density native treatment in oligotrophic or eutrophic conditions. Within the low‐density native species treatment, periphyton biomass was 71% lower in eutrophic conditions than in oligotrophic conditions. Within the invasive treatment, periphyton biomass was 57% lower in eutrophic conditions than in oligotrophic conditions. The other three species treatments were not different from the low‐density native treatment in oligotrophic or eutrophic conditions.

### 3.3 Zooplankton

We found a marginal interactive effect between the species treatment and trophic state on zooplankton (MANOVA: Pillai–Bartlet statistic = 0.6, *F*_9,72_ = 1.9, *p* = 0.064), but a stronger main effect of species treatment (MANOVA: Pillai–Bartlet statistic = 0.9, *F*_9,72_ = 3.2, *p* = 0.003). We followed up with a two‐way ANOVA to determine the individual responses of cladoceran, copepod and nauplii density to species treatment and trophic state. We only found rotifers in five mesocosms, so we were unable to draw any inferences about our experimental treatments on rotifers.

**FIGURE 5:**
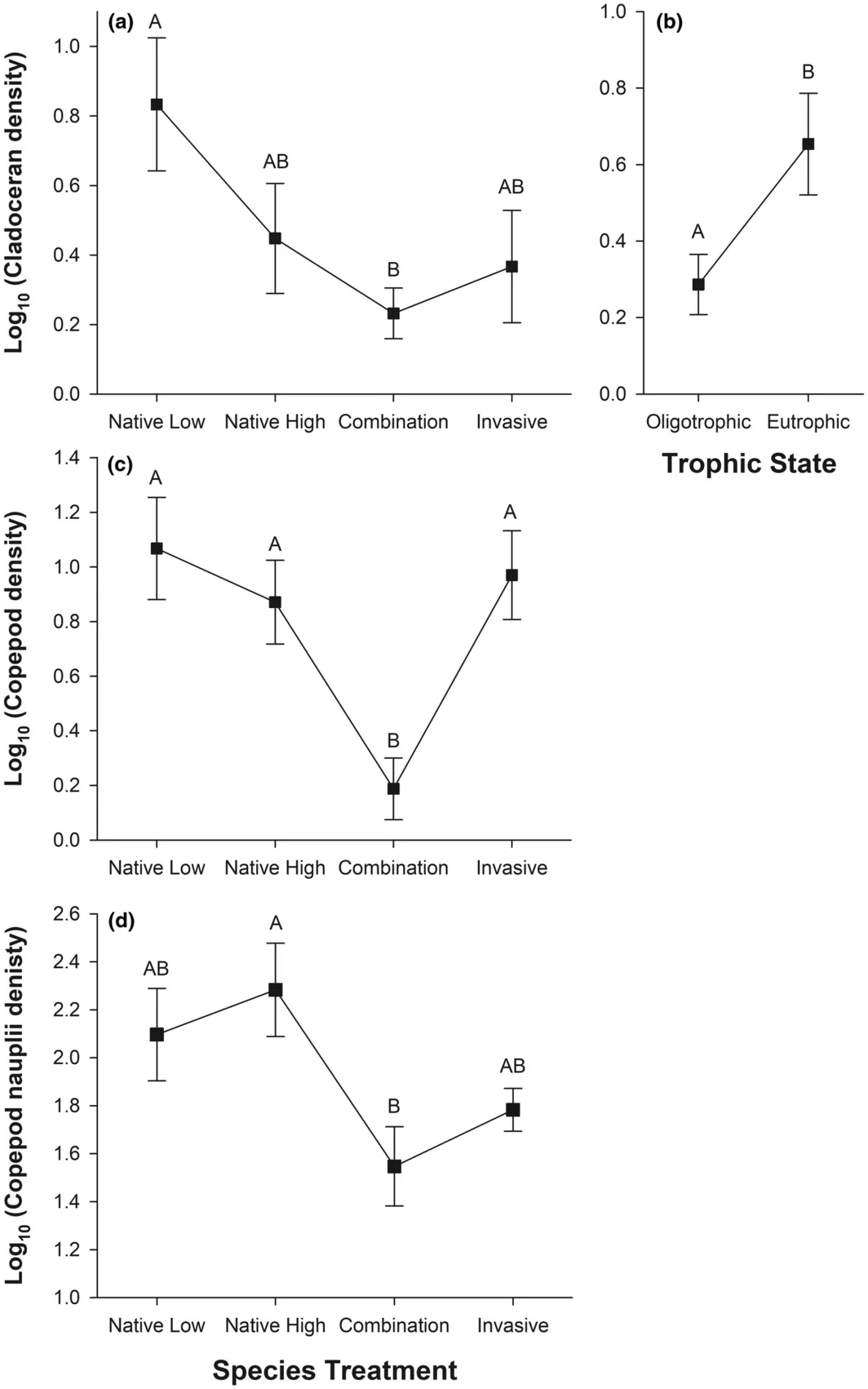
Main effects of species treatment (a) and trophic state (b) on the density (# of individuals/L) of cladoceran zooplankton, and the main effects of species treatment on adult copepod density (c; # of individuals/L) and copepod nauplii density (d; # of individuals/L). All error bars are ±1 SE. Letters above error bars indicate statistical differences where *p* < 0.05 for Holm‐Sidak pairwise comparisons.

We found no treatment interaction on cladoceran density (*F*_3,24_ = 1.6, *p* = 0.225), but there were main effects of species treatment (*F*_3,24_ = 3.7, *p* = 0.026) and nutrient conditions (*F*_1,24_ = 7.5, *p* = 0.012). Density was 92% lower in the combination treatment compared to low‐density natives (Figure 5a). Cladoceran density also was 81% lower in oligotrophic conditions than in eutrophic conditions (Figure 5b).

We found no treatment interaction (*F*_3,24_ = 1.4, *p* = 0.255) or a main effect of trophic state on copepod density (*F*_1,24_ = 0.08, *p* = 0.777), but there was a main effect of species treatment (*F*_3,24_ = 6.6, *p* = 0.002). The combination treatment had 89%–94% fewer copepods compared to the other species treatments (Figure 5c). Similarly, we found no treatment interaction (*F*_3,24_ = 2.8, *p* = 0.060) or a main effect of trophic state on copepod nauplii density (*F*_1,24_ = 0.06, *p* = 0.809). However, we found a main effect of species treatment (*F*_3,24_ = 4.5, *p* = 0.012). The combination treatment had 84% fewer nauplii compared to the high‐density native treatment (Figure 5d).

### 3.4 Macroinvertebrates

We found a marginal main effect of species treatment on macroinvertebrate responses (MANOVA: Pillai–Bartlet statistic = 0.7, *F*_12,69_ = 1.9, *p* = 0.053). We followed up with a one‐way ANOVA to determine the individual responses of the two amphipod species, snails and pea clams to species treatment.

**FIGURE 6:**
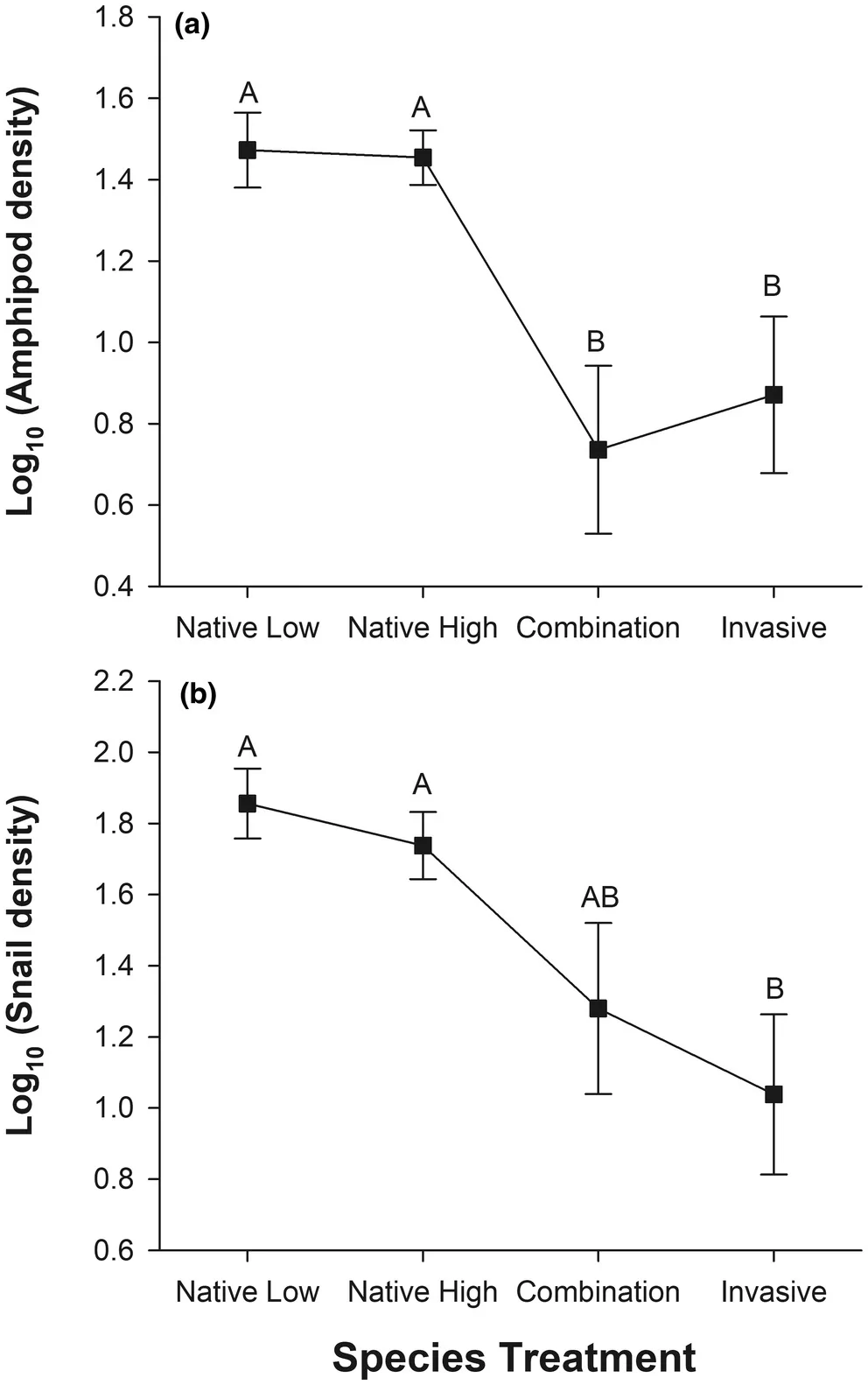
Main effects of species treatment on the density (# individuals/0.5 m^2^) of amphipods (a) and snails (b). All error bars are ±1 SE. Letters above error bars indicate statistical differences where *p* < 0.05 for Holm‐Sidak pairwise comparisons.

We found a main effect of species treatment on *Hyalella* amphipod density (*F*_3,28_ = 6.4, *p* = 0.002). *Hyalella* density was 66%–72% lower in the combination and invasive‐high treatments compared to both native‐species treatments (Figure 6a). We found no main effect of species treatment on the density of *Gammarus* amphipods (*F*_3,28_ = 2.2, *p* = 0.104).

We found a main effect of species treatment on *Physella* snail density (*F*_3,28_ = 4.7, *p* = 0.009). Snail density was 63%–72% lower in the invasive treatment than in the native species treatments (Figure 6b).

We observed no main effect of species treatment on the density of pea clams (*F*_3,28_ = 0.2, *p* = 0.867).

### 3.5 Native fish

**FIGURE 7:**
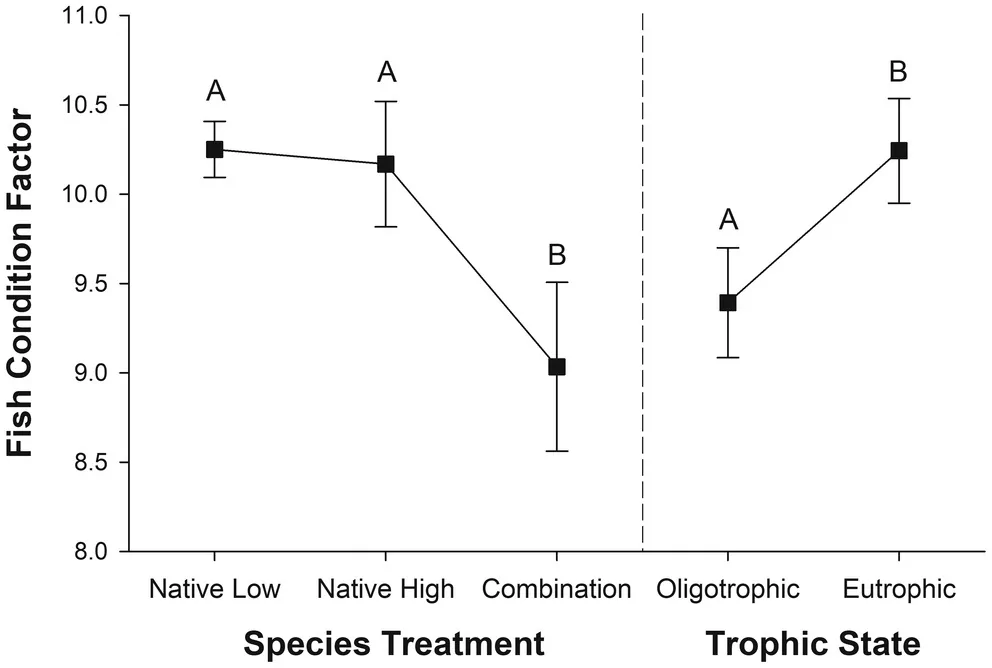
Main effects of species treatment (left) and nutrient treatment (right) on native fish condition. All error bars are ±1 SE. Letters above error bars indicate statistical differences where *p* < 0.05 for Holm‐Sidak pairwise comparisons.

We found no treatment interaction on native fish condition (*F*_2,18_ = 2.0, *p* = 0.164), but there were main effects of species treatment (*F*_2,18_ = 5.0, *p* = 0.019) and trophic state (*F*_1,18_ = 5.8, *p* = 0.027). Native fish condition was 12% lower in the combination treatment than in the low‐ and high‐density native fish treatments. Native fish condition also was 8% lower in oligotrophic conditions than in eutrophic conditions (Figure 7).

## 4 DISCUSSION

By using an additive and substitutive experimental design, we were able to determine whether the effects of adding goldfish were the result of adding more fish to the community versus adding an invasive fish to the community. From our 61‐day experiment, goldfish have the potential to trigger a rapid regime shift in lake ecosystems, especially under eutrophic conditions. Relative to the native‐fish treatments, this regime shift included increased suspended solids, reduced water clarity, less filamentous algae, fewer zooplankton, fewer macroinvertebrates and reduced native fish condition. Some of these responses depended on native fish density. Our results indicate that, regardless of whether a lake is oligotrophic or eutrophic, a goldfish invasion could lead to negative changes in water quality and ecological communities in lakes.

The goldfish‐induced reduction in water clarity under eutrophic conditions was likely the result of increased NVSS. The feeding method of the goldfish was probably the underlying mechanism that triggered more NVSS because they voraciously filter through sediments, consuming detritus, plant matter, benthic algae and invertebrates (Dickey et al., 2022; Hensley & Courtenay, 1980; Nico et al., 2025). This appears to be similar to what occurs when goldfish are added to fishless ponds (Lejeune et al., 2024). We did not find an interactive effect between species treatment and phytoplankton biomass. Moreover, the increase in phytoplankton was driven by adding native shiners or invasive goldfish (i.e. increasing from 3 to 6 fish), rather than from adding goldfish. Collectively, this suggests that changes in phytoplankton abundance were not responsible for the reduction in water clarity in our experiment despite this occurring in other studies (Lejeune et al., 2024).

While the higher oxygen concentrations in eutrophic conditions probably resulted from photosynthesis associated with higher phytoplankton abundance (Smith & Piedrahita, 1988), we found no evidence that goldfish caused declines in dissolved oxygen. Despite increases in NVSS and reductions in water clarity, our study suggests no threat of hypoxic conditions. However, our experimental ecosystem was only 0.5 m deep and we did not explore oxygen conditions at night. Future research could explore in situ oxygen dynamics of lakes where goldfish have been introduced, with measurements at greater depths and throughout the night to better understand this potential threat.

Phytoplankton and filamentous algae both increased under eutrophic conditions, which is a well‐known outcome of eutrophication (Brinson et al., 1981; Howarth et al., 2021; Smith, 1979). Across fish treatments, however, the increase in phytoplankton mirrored the decrease of filamentous algae among our species treatments. The reason for this mirrored effect is likely due to increased competition associated with increased density of either fish species alone or the two species combined. The diets of the golden shiner and goldfish are essentially the same, though feeding methods can be different (Hensley & Courtenay, 1980; Moyle, 2002; Murdy et al., 1997; Page & Burr, 2011). At higher densities, these two species therefore appeared to consume filamentous algae. Consumption, digestion and excretion likely lead to a feedback loop that fertilized phytoplankton production.

Alternative hypotheses for the mirrored changes in phytoplankton and filamentous algae includes a shading effect or an indirect effect through the zooplankton. The hypothesis that phytoplankton ‘shaded out’ filamentous algae through competition for light is less plausible because water clarity and NVSS were statistically the similar among the high‐native species and combination treatments. In addition, water clarity and NVSS did not change under oligotrophic conditions, yet the mirroring effect was present under oligotrophic conditions. Among the zooplankton groups, cladoceran density declined whenever we had six total fish (regardless of species composition), but the copepods exhibited more complex patterns. Given that cladocerans as a group are generally larger and more herbivorous than copepods in lakes (Sommer & Sommer, 2006), there is the potential that increased densities of native or invasive fish caused a reduction in cladocerans, which then caused an increase in phytoplankton via an indirect effect. This process would complement the increase in phytoplankton that would be caused by the fish consuming filamentous algae. Even though the large‐bodied cladocerans had lower densities in these fish treatments, statistical evidence was lacking to support a trophic cascade occurred. Ultimately, goldfish could trigger reductions in filamentous algae and more phytoplankton in lakes, but these effects largely depend on fish density and perhaps species composition or diet overlap within the native fish community.

The responses of the three zooplankton groups exhibited similar patterns in response to the fish treatments. From a strict interpretation of the statistical tests, only the combination of shiners and goldfish caused a decline in the cladocerans and copepods compared to the low‐density shiner treatment. This effect was the largest for copepods, which experience substantially lower abundance in the 3 + 3 combination compared to six shiners or six goldfish alone. Both fish species are known to consume microcrustaceans (Hensley & Courtenay, 1980; Moyle, 2002; Murdy et al., 1997; Page & Burr, 2011) and our results suggest that differences in foraging strategies between the shiners and goldfish might facilitate each other's predation, especially on copepods. In short, zooplankton communities might be more susceptible to fish predation where goldfish have invaded, but this might depend on the relative abundance of native versus invasive fish.

Goldfish led to a reduction in *Physella* snails and *Hyalella* amphipods, two of the four benthic macroinvertebrate species. We found very few pea clams across all mesocosms and no evidence of empty clam shells indicating poor survival, which suggests high rates of consumption by both fish species. Similarly, we found few *Gammarus* amphipods in the mesocosms, but we are unable to confirm if fish consumption or poor survival occurred since any dead individuals would have rapidly decomposed. Consumption by goldfish is a possible candidate for the reduction in density of *Physella* snails and *Hyalella* amphipods. However, the goldfish also cleared filamentous macroalgae, which would have depleted an important habitat and food source for macroinvertebrates. We did not have aquatic vegetation in our study, but Lejeune et al. (2024) found that goldfish introductions lead to the depletion of vegetation and the functional extinction of all taxa associated with the aquatic vegetation. Clearing of filamentous algae in our experiment by goldfish could have been one mechanism for the decline in snails and amphipods we observed. Periphyton biomass did not vary in a way that would have limited its availability as a food source to the snails and amphipods. We suspect that, by reducing filamentous macroalgae in the combination and invasive treatments, goldfish exposed macroinvertebrates to fish predation through habitat loss, which occurs with macrophytes in shallow lakes (Rennie & Jackson, 2005; Ziegler et al., 2017). It is also possible goldfish consumed the invertebrates due to their feeding mode and rate (Crone et al., 2023; Jugovic et al., 2025). This would explain the greater reduction in macroinvertebrates in treatments with goldfish compared to the high‐density native treatment where the biomass of filamentous algae was statistically comparable to the low‐density native treatments and those with goldfish. Similar to fishless shallow ponds (Lejeune et al., 2024), the shift towards a turbid state by goldfish in our experiment could result in lower macroinvertebrate diversity and abundance in lakes, and potentially food resources for some native fishes.

Native fish condition was similar at low and high densities but declined when combined with goldfish, clarifying that goldfish were responsible for the reduction in native fish condition in the combination treatment. This presumably occurred via exploitative competition, as suggested by the goldfish‐caused declines in benthic macroinvertebrates (e.g. see Dickey et al., 2022). Goldfish do not have a stomach and are known to have some of the highest feeding rates among fishes relative to size (Dickey et al., 2022). Where successful goldfish invasions occur, this indiscriminate and intense feeding rate could create intense exploitative competition with native fishes (Dickey et al., 2022; Massey et al., 2025). In fishless ponds, goldfish can exclude native predators (newts) over time through competition and food web collapse (Lejeune et al., 2024). While our short‐term experiment does not indicate our native fish predator would be excluded from the community, the reduction in native fish condition that we observed over 2 months might trigger substantial changes in growth and reproduction over longer time periods. We did not observe any agonistic behaviours in our experiment to suggest interference competition, but reduced water clarity in the invasive treatment would have prevented such observations. Nevertheless, fish size or condition is strongly associated with reproductive output (e.g. Barneche et al., 2018). Reductions in fish size could reduce reproductive output of native species where goldfish have invaded and compete with certain native species barring other ecological or environmental offsets.

Our experiment was a scaled down representation of a lake ecosystem and has limitations. Because a fish‐free control was not included, our experiment isolates the effects of fish identity and density but cannot distinguish them from the general impact of fish presence on ecosystem state. We also cannot know how long the regime shift we describe would persist or if hysteresis would occur if we removed goldfish. Furthermore, lakes are incredibly diverse, and we acknowledge that multiple processes in natural lakes could not be replicated. Some of our results are very similar to those that occurred in natural fishless ponds described by Lejeune et al. (2024). Even so, we are mindful that application of our results could be limited to smaller, shallower lakes containing fish. In these lakes, the patterns and processes we illustrate could also take more time than the duration of our experiment. A successful goldfish invasion would take time to achieve the density used in our experiment. Applying our results to larger deeper lakes is also uncertain. Large lakes are likely more resistant or resilient to the goldfish‐induced changes we found, which would also likely be limited to littoral areas in the photic zone. Nevertheless, Massey et al. (2025) warned that goldfishes are an increasing yet overlooked risk to freshwater ecosystems throughout North America. While our experiment has limitations, we hope our results reinforce this warning.

## 5 CONCLUSION

Our study illustrates that goldfish invasions could have negative consequences for water quality and ecological communities in lakes. Our results highlight the need for goldfish management. In addition to removal or control efforts, education and outreach to inform the public about the impacts of goldfish and why they should not be released is essential. Some of this information could be mandated to become part of the pet trade as well. It is important to acknowledge the value people place on pets, but it is critically important to inform customers of the pet trade that their pets can become pests. Clearly labelling or providing easy‐to‐understand literature to accompany all goldfish sales from the pet trade would help the public understand why we should not release these fish. Such information briefs could also identify humane methods of euthanasia. Human uses where goldfish are used as biological controls for algae also need to be regulated to ensure intentional or accidental release does not occur. There are numerous invaders of freshwater ecosystems. We encourage natural resource managers worldwide to add the goldfish to the list of invaders needing further investigation and management action.

## AUTHOR CONTRIBUTIONS

William D. Hintz and Rick A. Relyea conceived the ideas and designed the experiment; William D. Hintz and Hannah Barrett collected the data; William D. Hintz conducted the statistical analysis; William D. Hintz and Rick A. Relyea led the writing of the manuscript; all authors approved drafts for final publication.

## CONFLICT OF INTEREST STATEMENT

The authors declare no conflicts of interest.

## Data Availability

Data are available from the Figshare Digital Repository: 10.6084/m9.figshare.31062457 (Hintz et al., 2026).
